# Statin Eligibility according to 2013 ACC/AHA and USPSTF Guidelines among Jordanian Patients with Acute Myocardial Infarction: The Impact of Gender

**DOI:** 10.1155/2023/5561518

**Published:** 2023-06-05

**Authors:** Rashid Ibdah, Ahmad Alrawashdeh, Sukaina Rawashdeh, Nebras Y. Melhem, Ayman J. Hammoudeh, Mohamad I. Jarrah

**Affiliations:** ^1^Department of Internal Medicine, Faculty of Medicine, Jordan University of Science and Technology, Irbid 22110, Jordan; ^2^Department of Allied Medical Sciences, Jordan University of Science and Technology, Irbid 22110, Jordan; ^3^Department of Anatomy, Physiology and Biochemistry, Faculty of Medicine, The Hashemite University, Zarqa 13115, Jordan; ^4^Department of Cardiology and Coronary Computed Tomography Section, Istishari Hospital, Amman, Jordan

## Abstract

The objectives of this study were to evaluate statin eligibility among Middle Eastern patients admitted with acute myocardial infarction (AMI) who had no prior use of statin therapy, according to 2013 ACC/AHA and 2016 USPSTF guidelines, and to compare statin eligibility between men and women. This was a retrospective multicenter observational study of all adult patients admitted to five tertiary care centers in Jordan with a first-time AMI, no prior cardiovascular disease, and no prior statin use between April 2018 and June 2019. Ten-year atherosclerotic cardiovascular disease (ASCVD) risk score was estimated based on ACC/AHA risk score. A total of 774 patients met the inclusion criteria. The mean age was 55 years (SD ± 11.3), 120 (15.5%) were women, and 688 (88.9%) had at least one risk factor of cardiovascular disease. Compared to men, women were more likely to be older; had a history of diabetes, hypertension, and hypercholesterolemia; and had higher body mass index, systolic blood pressure, total cholesterol, and high-density lipoproteins. Compared to women, men were more likely to have a higher 10-year ASCVD risk score (14.0% vs. 17.8%, *p* = 0.005), and more men had a 10-year ASCVD risk score of ≥7.5% and ≥10%. The proportion of patients eligible for statin therapy was 80.2% based on the 2013 ACC/AHA guidelines and 59.5% based on the USPSTF guidelines. A higher proportion of men were eligible for statin therapy compared to women, based on both the 2013 ACC/AHA (81.4% vs. 73.5%, *p* = 0.050) and USPSTF guidelines (62.0% vs. 45.2%, *p* = 0.001). Among Middle Easterners, over half of patients with AMI would have been eligible for statin therapy prior to admission based on the 2013 ACC/AHA and USPSTF guidelines, with the presence of gender gap. Adopting these guidelines in clinical practice might positively impact primary cardiovascular preventive strategies in this region.

## 1. Introduction

Cardiovascular diseases (CVDs) remain among the leading causes of death worldwide, accounting for over 30% of global deaths in 2019 [1, 2]. The use of statins has reduced the mortality and morbidity associated with CVDs worldwide [3]. However, underprescription of statins is a serious issue in the Middle East. For example, statins were recently reported as the most underprescribed medications among older Lebanese patients [4]. Further studies are essential to understand the eligibility of statin therapy in the region, particularly among patients with CVDs.

Cholesterol-lowering drugs, statins, are the first-choice treatments for secondary prevention of atherosclerotic cardiovascular diseases (ASCVD) [5]. The American College of Cardiology (ACC) and the American Heart Association (AHA) released a list of guidelines in 2013 and 2019 that define the doses and eligibility for statin treatment to manage CVD risk in adults. The guidelines identify high- and moderate-intensity statin therapy for use in primary and secondary prevention [6, 7]. In 2016, the US Preventive Services Task Force (USPSTF) released recommendations on statin therapy for the primary prevention of ASCVD [8]. According to these guidelines, individuals aged 40 to 75 years and with one or more ASCVD risk factors (hypertension, tobacco use, diabetes mellitus (DM), or dyslipidaemia) and a ten-year ASCVD risk ≥ 10% are advised to start statin therapy. The AHA/ACC and USPSTF guidelines differ in grading and assigning levels of evidence and classes of recommendations at a population level [9].

Lipid-lowering therapies are essential for the primary and secondary prevention of ASCVD [10]. Statin therapy in patients aged more than 65 years old decreases the risk of major cardiovascular events by 19% [11]. However, statin underprescription is a widespread issue, specifically among older populations [12–15]. Furthermore, prescription of statins has been shown to be affected by gender discrepancies [16, 17]. Information is scarce concerning what clinical practice has been established among patients with CVDs in regard to statin therapy in Jordan. Therefore, the present study is aimed at evaluating statin eligibility based on the 2013 ACC/AHA and USPSTF 2016 guidelines for the treatment of blood cholesterol in a cohort of adults who experienced first-time acute myocardial infarction (AMI) and to investigate potential gender impact in statin eligibility.

## 2. Materials and Methods

### 2.1. Study Design and Setting

This was a retrospective multicenter study involving all patients presented with a first-time AMI with no prior statin use, across five tertiary care centers in Jordan between April 2018 and June 2019. The ethical approval to conduct the study was obtained from the Institutional Review Board of the King Abdullah University Hospital (IRB no. 53/114/2018). Ethical approval was obtained from all participating hospitals. This study was conducted in compliance with the ethical standards per Helsinki Declaration. The included patients agreed to participate and provided written informed consent with a cover letter containing a description of the study, the participant's ethical and legal rights, and the researcher's contact information which were provided for each participant. The Strengthening the Reporting of Observational Studies in Epidemiology (STROBE) cross-sectional reporting guidelines were used in our study [18].

### 2.2. Study Participants and Data Collection

The included patients were screened and enrolled to the study by trained medical students who received training for the inclusion criteria and data collection. To be enrolled in this study, patients must present with ST-elevation myocardial infarction (STEMI) and non-ST-elevation myocardial infarction (NSTEMI) for first time with no prior cardiovascular diseases and with no present or past use of statin. All participants were confirmed to have had their lipid profile measured upon admission. Patients with prior statin use and those without lipid profile data were excluded from the study.

The data regarding lipid profile, blood pressure, body mass index, and electrocardiography (ECG) were collected upon hospital admission. The body mass index (BMI) was calculated as weight/height^2^ (kg/m^2^). The remaining parameters were collected from the patients' medical records upon admission. In addition, other demographic and risk factors included age and gender, diabetes mellitus (DM), hypertension, hypercholesterolemia, smoking, and family history of cardiovascular diseases, which were collected during the interview at admission. The diagnosis of AMI was confirmed by the cardiologist in each participating hospital. During the enrolment period, no further medical complications were detected among the participants. Blood samples were taken for lipid and glucose analysis. Each patient was monitored carefully during the procedure by medical residents.

Ten-year ASCVD risk was estimated based on ACC/AHA risk score (2013) for the majority of the patients (84.9%) [19]. The ASCVD risk score for a total of 117 patients could not be estimated because the value of the parameters included in the ACC/AHA equation was not within the allowed range (i.e., age (20-79), systolic blood pressure (SBP) (90-130), total cholesterol (TC) (130-320), and high-density lipoprotein cholesterol (HDL-C) (20-100)). To manage missing value for those patients, we estimated the 10-year ASCVD risk score using the Framingham 2008 formula [20]. However, the 10-year ASCVD risk score eventually could not be estimated for a total of 17 patients, for the same reason above. The 10-year ASCVD risk scores were estimated using the CV risk R package [21]. The 2013 ACC/AHA and 2016 USPSTF guidelines were used to identify statin eligibility.

### 2.3. Statistical Analysis

For descriptive analysis, continuous data were presented as means and standard deviations (SDs) or medians and interquartile ranges (IQR), and categorical variables were presented as frequency and percentages (%). Percentages were compared between both genders using chi-square test, while means or medians were compared using the Student *t*-test or Mann–Whitney test as appropriate. Binary logistic regression was used to estimate the association between statin eligibility for both the 2013 ACC/AHA and USPSTF guidelines and gender, without adjusting for other factors. The association was presented as unadjusted odds ratio (OR). A *p* value of <0.050 was considered as statistically significant. All assumptions of statistical procedures were assured and met. Data analysis was performed using Stata software package (Stata Corp 16).

## 3. Results and Discussion

### 3.1. Study Participants

Of 774 patients who met the inclusion criteria, 548 (70.8%) had STEMI and 226 (29.2%) had NSTEMI. The mean age was 55 years (SD ± 11.3), and 120 (15.5%) were women and 292 (37.7%) had DM. Almost 89% (*n* = 688) had at least one risk factor of CVDs and 61.2% (*n* = 474) were active smokers.

Participants' characteristics and the association between demographic and clinical characteristics of the study population and gender are presented in Table 1. Compared to men, women were older and were more likely to have a history of DM, hypertension, and hypercholesterolemia, but less likely to be smokers (*p* < 0.001). Women had a higher level of BMI, SBP, diastolic blood pressure (DBP), TC, and HDL-C (*p* < 0.050). Although there was no statistical difference in the type of AMI between men and women, women were less likely to receive percutaneous coronary intervention (PCI) and more likely to receive coronary artery bypass grafting (CABG).

### 3.2. The 10-Year ASCVD Risk Score

The description of the 10-year ASCVD risk score for our sample is presented in Table 2. The mean 10-year ASCVD risk score of the total population was 17.2% (SD ± 13.5). Men had higher mean 10-year ASCVD risk score compared to women (mean = 17.8 vs. 13.9, *p* = 0.005). In addition, compared to women, men were more likely to have a 10-year ASCVD risk score of ≥7.5% (54.7% vs. 78.1%, *p* < 0.001) and a 10-year ASCVD risk score of ≥10% (47.8% vs. 67.9%, *p* < 0.001).

### 3.3. Statin Eligibility and Gender Gap

Table 3 shows the proportion of total patients and both genders who were eligible for statin therapy by both the 2013 ACC/AHA and USPSTF guidelines as well as the results of univariable logistic regression for both genders. According to the 2013 ACC/AHA guidelines for statin eligibility, a total of 610 (80.1%) patients were eligible for statin therapy. Compared to women, there was weak evidence that men were more likely (73.5% vs. 81.3%, *p* = 0.050) and had higher odds (OR = 1.57; 95% CI 1.00-2.48) to be eligible for statin therapy. However, women were more likely and had higher odds (OR = 2.06; 95% CI 1.38-3.06) to be eligible for statin based on the second category (DM patients aged 40-75 with LDL-C ranged between 70 and 189), while men were more likely and had higher odds (OR = 2.96; 95% CI 1.96-4.47) to be eligible for statin therapy based on the third category (10-year ASCVD risk ≥ 7.5) of the 2013 ACC/AHA guideline.

According to the USPSTF guideline, a total of 450 (59.4%) patients were eligible for statin therapy. Compared to women, men were more likely (45.2% vs. 61.9%, *p* = 0.001, respectively) and had higher odds (OR = 1.98; 95% CI 1.32-2.95) to be eligible for statin therapy. The only category of the USPSTF guideline that showed significant difference between men and women was the 10-year ASCVD risk score of ≥10%.

## 4. Discussion

In the present multicenter study, we evaluated the statin eligibility based on the 2013 ACC/AHA and USPSTF 2016 guidelines in a cohort of adults who experienced a first-time AMI in a patient cohort in Jordan. Our findings indicated that 60% to 80% of our patients were eligible for statin therapy. Compared to the USPSTF guidelines, the 2013 ACC/AHA guidelines identified more eligible patients by 35%. In addition, compared to women, men were more likely to have a higher eligibility for statin therapy based on both the 2013 ACC/AHA and USPSTF guidelines.

A total of 774 patients met the inclusion criteria. The mean age was 55 years (SD ± 11.3), 120 (15.5%) were women, and 688 (88.9%) had at least one risk factor of cardiovascular disease. Compared to men, women were more likely to be older; had a history of DM, hypertension, and hypercholesterolemia; and had a higher level of BMI, systolic blood pressure, total cholesterol, and high-density lipoproteins. Compared to women, men were more likely to have a higher 10-year ASCVD risk score (45.2% vs. 62.0%, *p* = 0.001), and more men had a 10-year ASCVD risk score of ≥7.5% and ≥10%. The proportion of patients who were eligible for statin therapy was 80.2% based on the 2013 ACC/AHA guidelines and 59.5% based on the USPSTF guidelines. A higher proportion of men were eligible for statin therapy compared to women, based on both the 2013 ACC/AHA and USPSTF guidelines.

However, we observed that more women were eligible for statin therapy based on the second category of AHA guidelines than men. This is likely associated with the higher likelihood of women to be older and diabetic among the population. Specifically, women displayed elevated body mass index (BMI), blood pressure levels, LDL-C, and hypercholesterolemia. Consistent with our findings, previous studies have reported similar associations. Numerous clinical trials have consistently revealed that female patients diagnosed with AMI tend to be of advanced age and exhibit higher prevalence of diabetes mellitus, hypertension, and other associated complications [22–26]. These findings are of significant importance, as they elucidate a constellation of factors that contribute to the increased susceptibility of this population to DM.

In 2013, the ACC/AHA framed certain guidelines on the management of the major risk factor for ASCVD, i.e., blood cholesterol levels, to reduce the risks for ASCVD in the adult population. These recommendations were based on the random clinical trials (RCTs) and applied to both males and females [27]. Reaching a target cholesterol level is not a part of the updated guidelines. The updated guidelines recommend an appropriate dose of statin for primary prevention that is based on an individual's ASCVD risk scores, or comorbidities like diabetes or elevated blood LDL-C levels. These guidelines advocate the use of statin therapy and are not based on achieving LDL-C targets; rather, they emphasize on LDL-C lowering [28].

It is reported that the USPSTF statin guidelines resulted in a 15% decrease in statin eligibility when compared to the 2013 ACC/AHA guidelines in the atherosclerosis patients who were not on statin therapy [29]. Consistent with these findings in the present study also, we observed a significantly higher number of patients ineligible for statin therapy according to the USPSTF guidelines as compared to the 2013 ACC/AHA guidelines. Patients found ineligible by the USPSTF had a higher ASCVD risk as compared to those found ineligible by the ACC/AHA. These findings highlight the fact that the health care providers or clinicians should wisely choose the guidelines to determine the eligibility of the patients for statin therapy that may impact the ASCVD outcomes.

In a study comprising of 4854 people, 66% of women were found to be eligible for statin therapy as per the ACC/AHA guidelines. This study also reported the overestimation of ASCVD risk [30]. In the present study, we also found the majority of men to be statin eligible as per the ACC/AHA and USPSTF guidelines. In another multiethnic study, a 28% increase in statin eligibility was observed by the ACC/AHA guidelines compared with the ATP-III guidelines [31].

The prevalence of morbidity and mortality after first MI is more prevalent in women compared to men [32]. Among the survivors of MI, the risk of recurrence of MI and heart failure is more in women as compared to men [33]. It is reported that the one-year mortality post-MI was 44% in women as compared to 27% in men. The short-term and long-term mortality post-MI is reported to be about 40% higher in women as compared to men [34]. Regardless of a higher risk of MI and higher morbidity and mortality in women, only half of the women as compared to men are treated with thrombolytic therapies. The mortality due to MI was found to be double in women less than 50 years as compared to men in the same age group [35].

It has been seen in CVD cases that men receive more frequent treatment as compared to women. The poor prognosis in women, as evident from several clinical studies, may be due to the fact that CVDs occur lately in women as compared to men (approximately 7-10 years). According to the USAGE study [36], women are also at a higher risk of statin noncompliance than men and are more likely to stop or switch their statin therapy because of side effects than men. Another explanation for the poor prognosis in women may be the prevalence of other comorbidities such as diabetes and chronic kidney diseases. These facts together complicate the process of the management of CVDs in women [37]. Consistent with these findings, in the present study, also, we observed that a significantly higher proportion of women was eligible for statin therapy as compared to the men. Undertreatment of women in primary and secondary prevention of CVDs may be influenced by the misconception of CVDs as a predominantly male problem [38]. Furthermore, this disparity may be related to ill-defined risk stratification or underutilization of guidelines in women compared to men.

However, according to the ASCVD risk based on the 2013 ACC/AHA and 2016 USPSTF guidelines, men were more likely to be eligible for statin therapy. It is documented that there is a strong relationship between prevalence of CVD risk factors and lifestyle. Smoking, for example, increases the risk of developing ASCVD [39]. Our findings may be corroborated by the staggeringly high rate of Jordanian male smokers (54.9%) compared to females (8.3%) [40].

It is documented that statins are equally effective in men and women [41, 42]. In a meta-analysis that analysed the effect of all lipid-lowering therapies from 1996 to 2003, it was concluded that statins significantly reduced CVD events, mortality, and MI in women [43]. However, it is difficult to confirm the effectiveness of statin therapy in women with CVDs because of the fact that most of the studies related to statin therapy have included very few women. Studies that included more women did not provide results by gender classification; rather, they provide conclusions based on the primary outcomes in general. Another study showed that statins reduced the risk of CVD events in both men and women. However, it was concluded that women on statin therapy might not have reductions in mortality and stroke like their male counterparts [44]. In a meta-analysis that included 11,435 women, it was observed that statin therapy did not show any better outcome in terms of CVD events and mortality as compared to placebo [43]. These observations warrant more studies, including a higher number of women with CVD events to confirm the gender-specific statin response in managing CVDs.

The primary reason for the underprescription of statins in elderly coronary patients is the perceived lack of indication, which stresses the need of extensive guidelines for prescription in elderly patients. Despite recommendations issued in international and national guidelines, the use of lipid-lowering medications in Jordan remains suboptimal.

## 5. Limitations

The results of this study should be interpreted with caution. First, this was a cross-sectional descriptive design which may limit the external validity of the results. Second, selection bias is potential as the results were generated on patients who had first-time AMI. Thus, the results may not be generalized to the total population of the Middle East. A larger study with more comprehensive inclusion criteria and using experimental longitudinal design may be necessary to further understand the eligibility of statin therapy among Middle Easterners. Also, the impact of confounders has not been assessed as we have not adjusted for other factors when estimating the odds ratio. However, most factors have been involved in estimating the 10-year ASCVD risk. Finally, the application of the 2013 ACC/AHA guidelines might be outdated, and future studies should consider the application of the 2019 ACC/AHA guidelines.

## 6. Conclusions

In this Middle Eastern study of patients with first-time AMI, evaluation for statin eligibility based on the 2013 ACC/AHA and USPSTF guidelines showed that a major proportion of individuals would have been eligible for statin therapy prior to presentation. We observed a gender impact in the statin eligibility in the study cohort. Men were more likely and had higher odds to be eligible for statin therapy as compared to women. Adopting these guidelines in clinical practice might positively impact primary cardiovascular preventive strategies in this region.

## Figures and Tables

**Table 1:** The association between demographic and clinical characteristics of the study population and gender.

	Total n =774	Men n =654	Women n =120	*p* value
Age (years), mean ± SD	55.0 (11.3)	54.3 (11.2)	58.58 (11.2)	<0.001
Caucasian	772 (99.7)	652 (84.4)	120 (15.5)	0.544
CVD risk factor				
Diabetes	292 (37.7)	224 (34.2)	68 (56.6)	<0.001
Hypertension	316 (40.8)	244 (37.3)	72 (60.0)	<0.001
Hypercholesterolemia	189 (24.4)	143 (21.8)	46 (38.3)	<0.001
Smoking	474 (61.2)	446 (68.2)	28 (23.3)	<0.001
Had ≥1 CVD risk factor	688 (88.9)	586 (89.6)	102 (85.0)	0.143
Family history of CVDs	320 (41.3)	269 (41.1)	51 (42.5)	0.780
BMI (kg/m^2^), mean ± SD	28.3 (4.7)	28.1 (4.4)	29.7 (5.7)	<0.001
Systolic BP (mmHg), mean ± SD	129.0 (21.0)	128.0 (20.3)	134.9 (23.3)	<0.001
Diastolic BP (mmHg), mean ± SD	77.5 (11.8)	77.1 (11.6)	79.7 (12.8)	0.030
TC (mg/dL), mean ± SD	187.5 (52.1)	186.0 (50.1)	196.1 (61.1)	0.049
LDL-C (mg/dL), mean ± SD	125.7 (46.0)	124.7 (44.1)	131.2 (54.7)	0.155
HDL-C (mg/dL), mean ± SD	37.3 (10.8)	36.4 (9.7)	42.3 (14.5)	<0.001
TG (mg/dL), median (IQR)	156 (110-222)	155 (108-221)	159 (115-235)	0.365^∗^
Type of AMI				
STEMI	548 (70.8)	467 (71.4)	81 (67.5)	0.387
NSTEMI	226 (29.2)	187 (28.5)	39 (32.5)	0.387
Intervention				
Cath	752 (97.1)	636 (97.2)	116 (96.6)	0.725
PCI	720 (93.0)	614 (93.8)	106 (88.3)	0.028
CABG	7 (0.9)	4 (0.6)	3 (2.5)	0.045
Statin given after AMI	746 (96.3)	629 (96.1)	117 (97.5)	0.476

Results are presented as frequency (%), unless otherwise indicated. Except for TG, all continuous variables were normally distributed. The differences between genders were assessed using the Student *t*-tests for means and chi-square tests for percentages. ^∗^Mann–Whitney test. CVD: cardiovascular disease; BMI: body mass index; BP: blood pressure; TC: total cholesterol; LDL-C: low-density lipoprotein cholesterol; HDL-C: high-density lipoprotein cholesterol; TG: triglycerides; AMI: acute myocardial infarction; STEMI: ST-elevation myocardial infarction; NSTEMI: non-ST-elevation myocardial infarction; PCI: percutaneous coronary intervention; CABG: coronary artery bypass graft.

**Table 2:** The 10-year ASCVD risk score to the total sample and differences between genders.

10-year ASCVD risk score	Total	Men	Women	*p* value^∗^
Risk score, mean (SD)	17.2 (13.5)	17.8 (13.5)	13.9 (13.4)	0.005
<5	111 (14.6)	77 (12.0)	34 (29.5)	<0.001
5 to <7.5	81 (10.7)	63 (9.8)	18 (15.6)	0.062
7.5 to <10	74 (9.7)	66 (10.2)	8 (6.9)	0.341
≥7.5%	565 (74.6)	502 (78.1)	63 (54.7)	<0.001
≥10%	491 (64.8)	436 (67.9)	55 (47.8)	<0.001

Results are presented as frequency (%), unless otherwise indicated. Missing 13 (0.02%). ^∗^Chi-square test. ASCVD: atherosclerotic cardiovascular disease.

**Table 3:** Statin eligibility by the 2013 ACC/AHA and USPSTF guidelines.

	Total	Men		Women		*p* value	*M* ^∗^
	*n* (%)	*n* (%)	OR (95% CI)	*n* (%)	OR (95% CI)		
2013 ACC/AHA guidelines							
Statin eligible overall	610 (80.1)	524 (81.3)	1.57 (1.00-2.48)	86 (73.5)	0.64 (0.40-1.00)	0.050	13
LDL − C ≥ 190	61 (7.8)	49 (7.4)	0.73 (0.38-1.42)	12 (10.0)	1.37 (0.71-2.66)	0.353	0
DM patients aged 40-75 with LDL-C (70-189)	240 (31.0)	186 (28.4)	0.49 (0.33-0.72)	54 (45.0)	2.06 (1.38-3.06)	<0.001	0
10-year ASCVD risk ≥ 7.5%	565 (74.6)	502 (78.1)	2.96 (1.96-4.47)	63 (54.7)	0.34 (0.22-0.51)	<0.001	17

USPSTF guidelines							
Statin eligible overall	450 (59.4)	398 (61.9)	1.98 (1.32-2.95)	52 (45.2)	0.51 (0.34-0.76)	0.001	17
Aged between 40 and 75	689 (89.0)	583 (89.1)	1.08 (0.59-1.99)	106 (88.3)	0.92 (0.50-1.70)	0.778	0
Had ≥1 CVD risk factor	688 (88.8)	586 (89.6)	1.52 (0.87-2.66)	102 (85.0)	0.66 (0.38-1.15)	0.141	0
10-year ASCVD risk ≥ 10%	491 (64.8)	436 (67.9)	2.31 (1.55-3.45)	55 (47.8)	0.43 (0.29-0.65)	<0.001	17

Univariable binary logistic regression was used to estimate unadjusted odds ratios. ^∗^Missing values. unadjusted odds ratio:OR; ACC/AHA: American College of Cardiology/American Heart Association; CVDs: cardiovascular disease; LDL-C: low-density lipoprotein cholesterol; DM: diabetes mellitus; ASCVD: atherosclerotic cardiovascular disease; USPSTF: US Preventive Services Task Force.

## Data Availability

The datasets generated and/or analysed during the current study are available from the first or corresponding author on reasonable request.

## Additional Points

*Data Sharing Statement*. Data might be shared after a formal approval from the participating hospitals.
